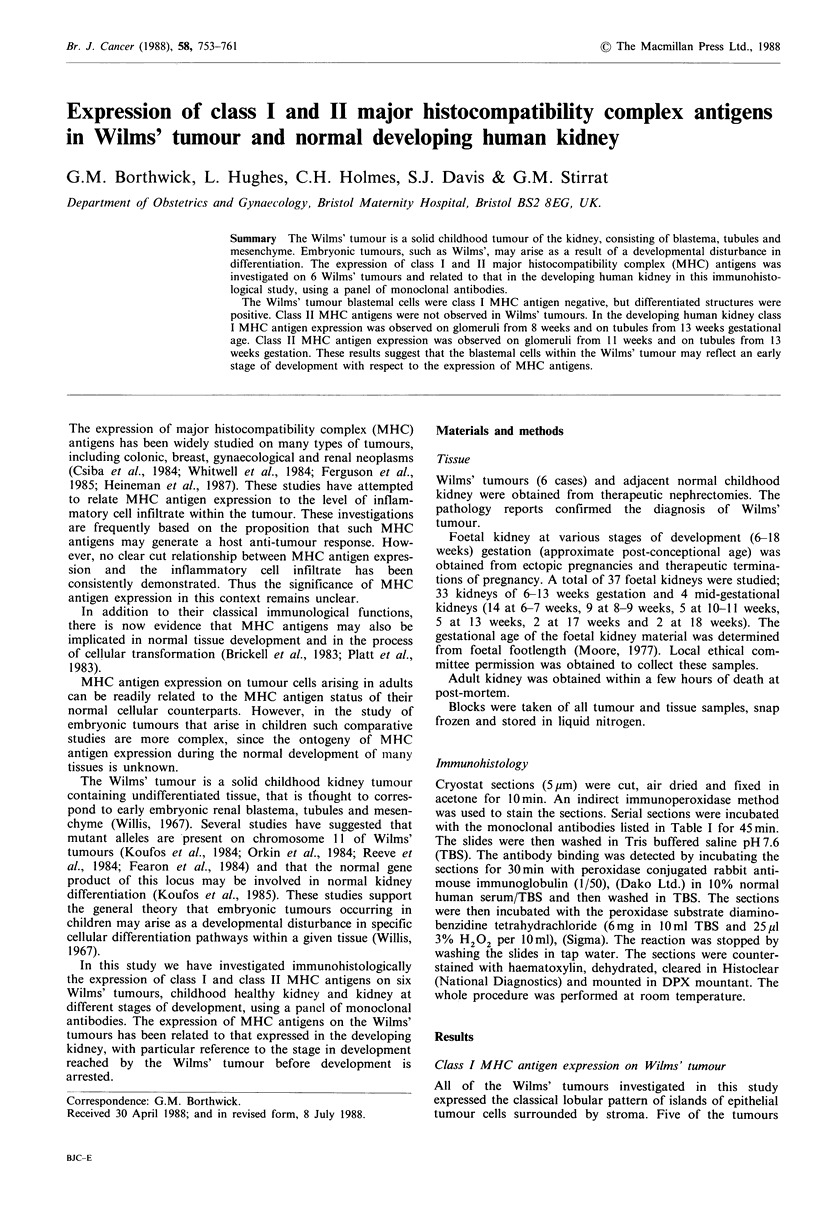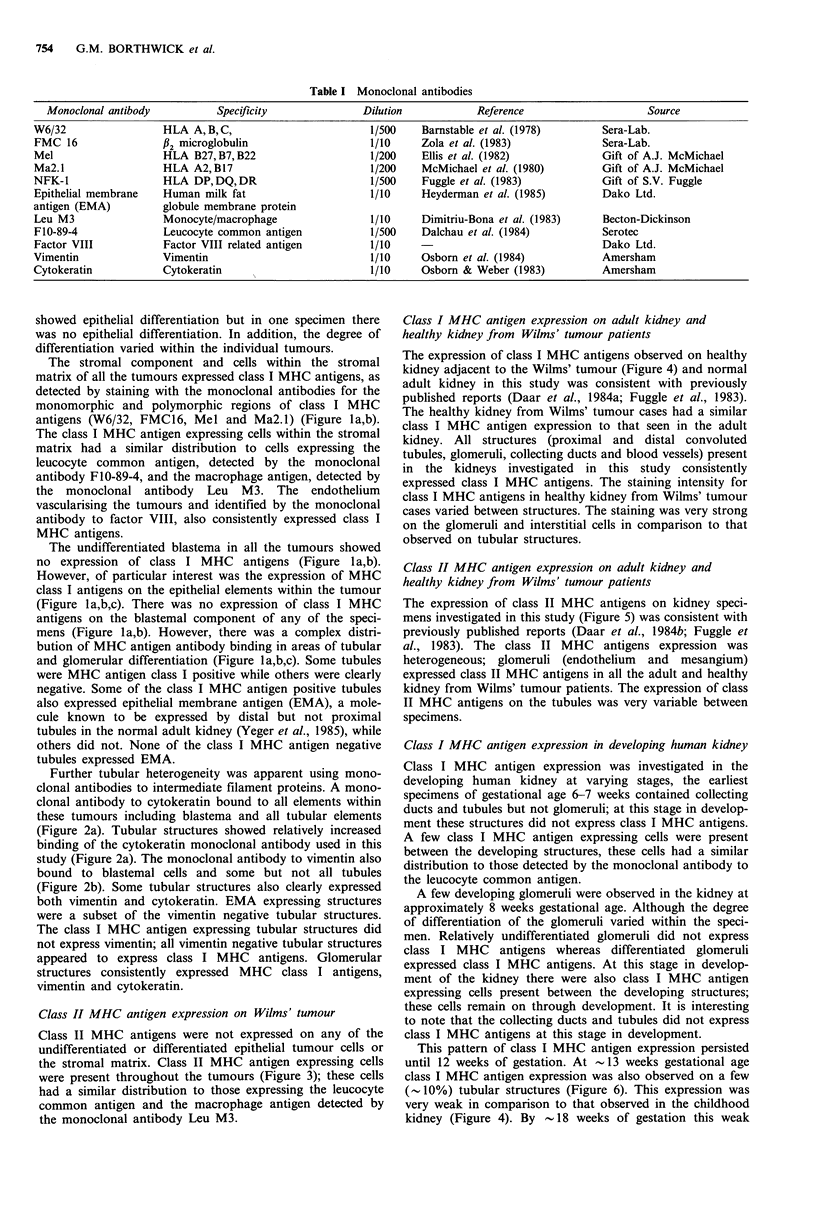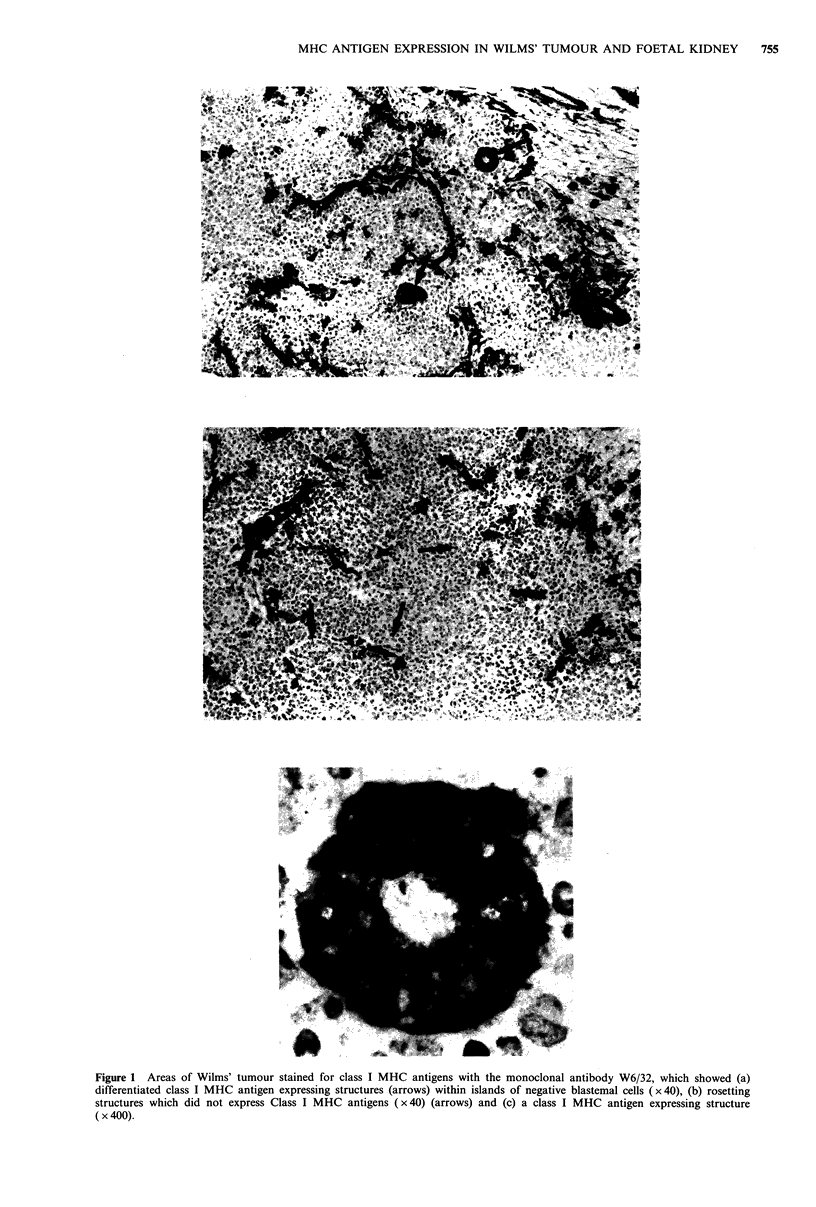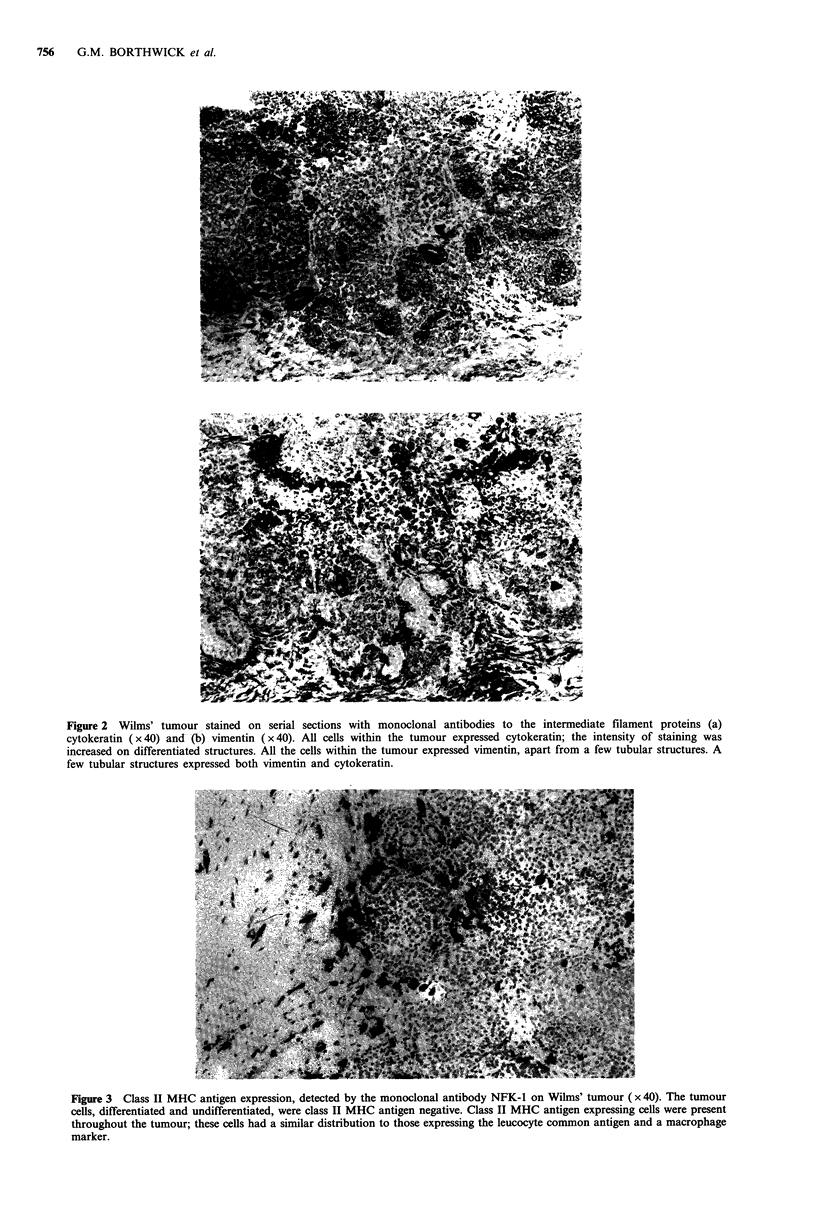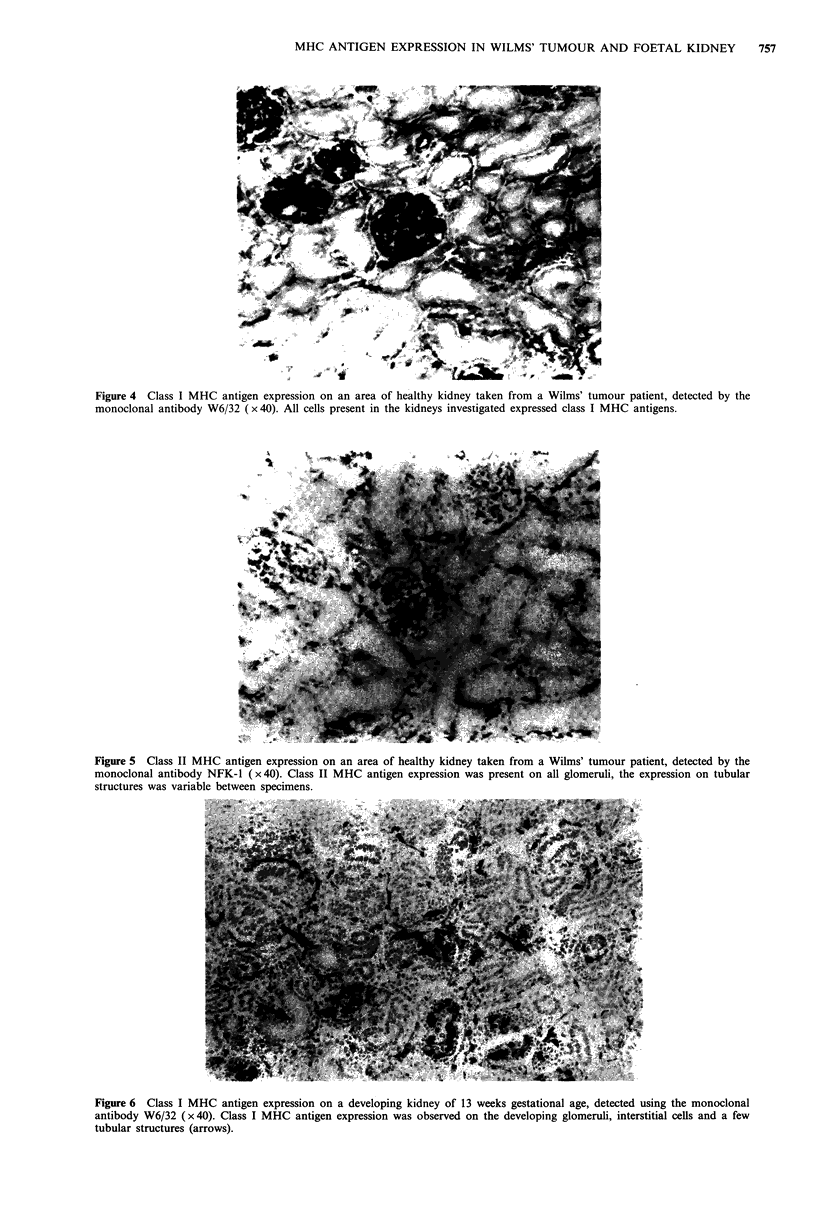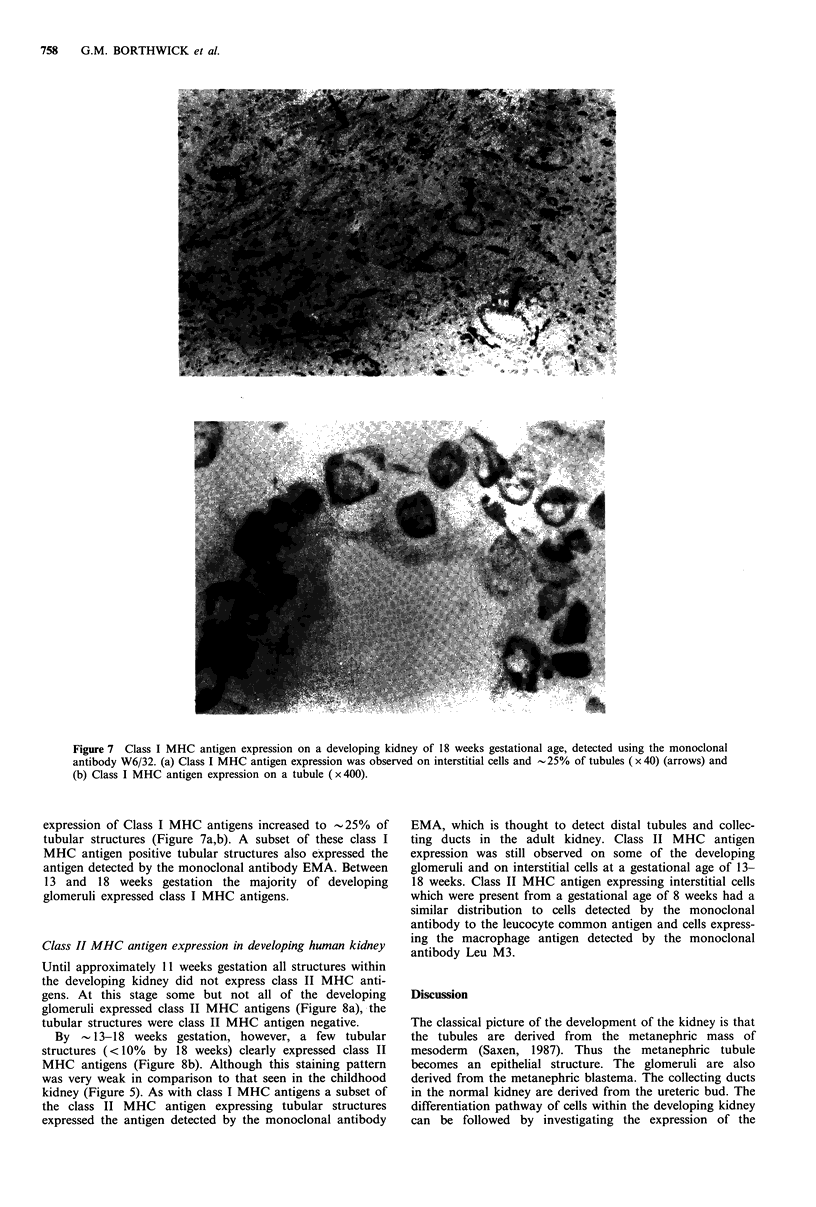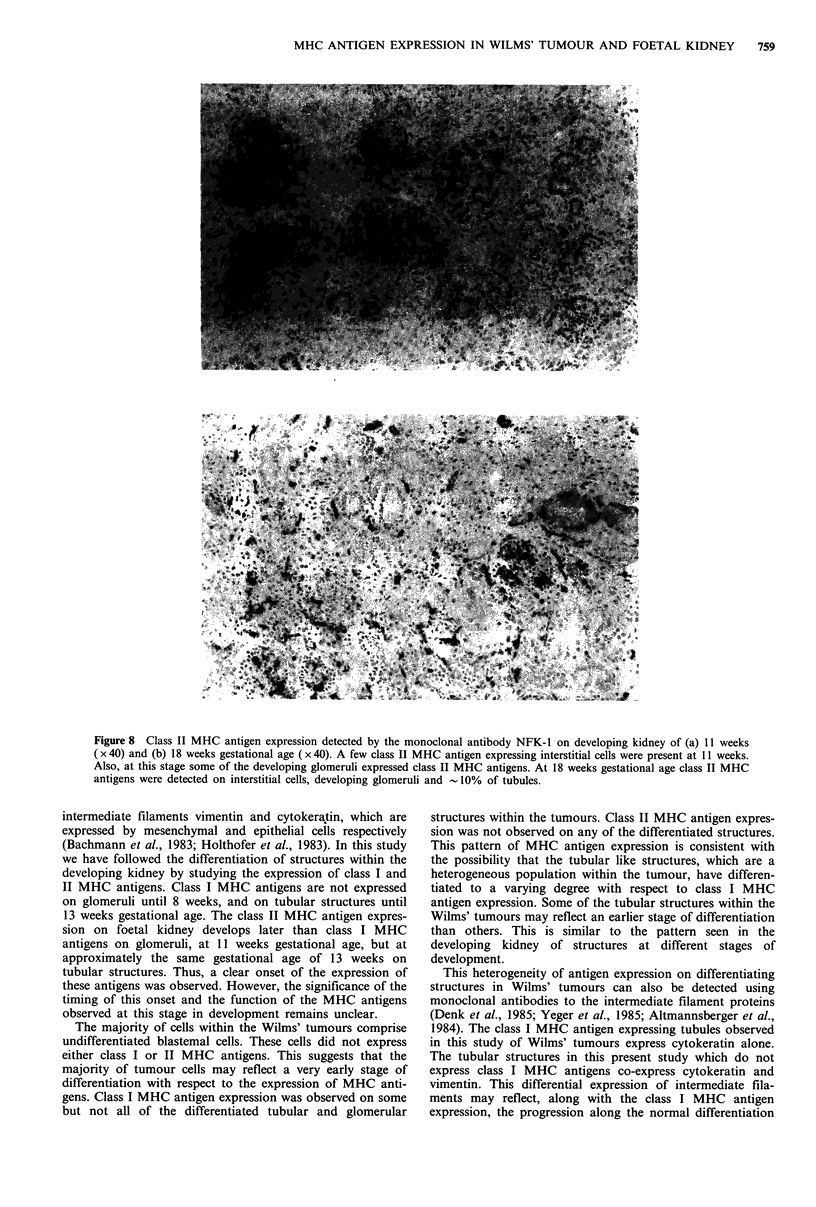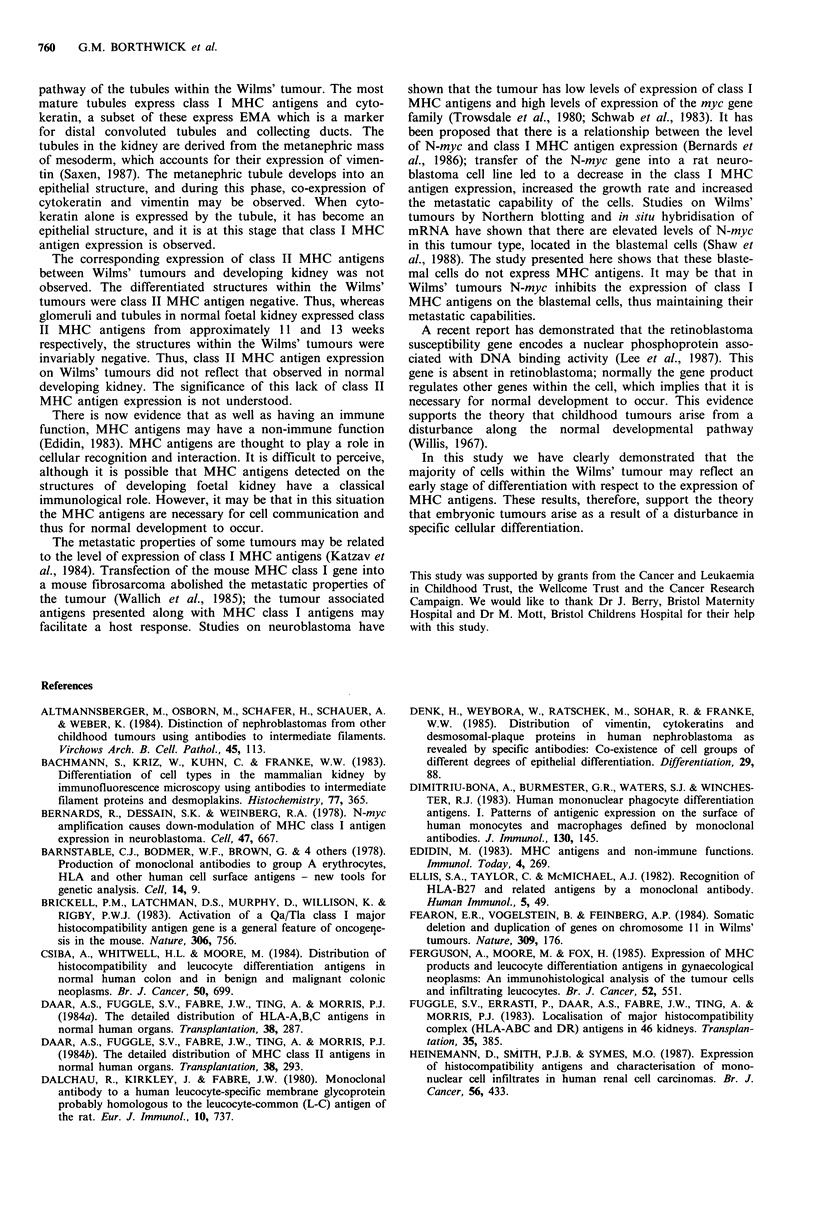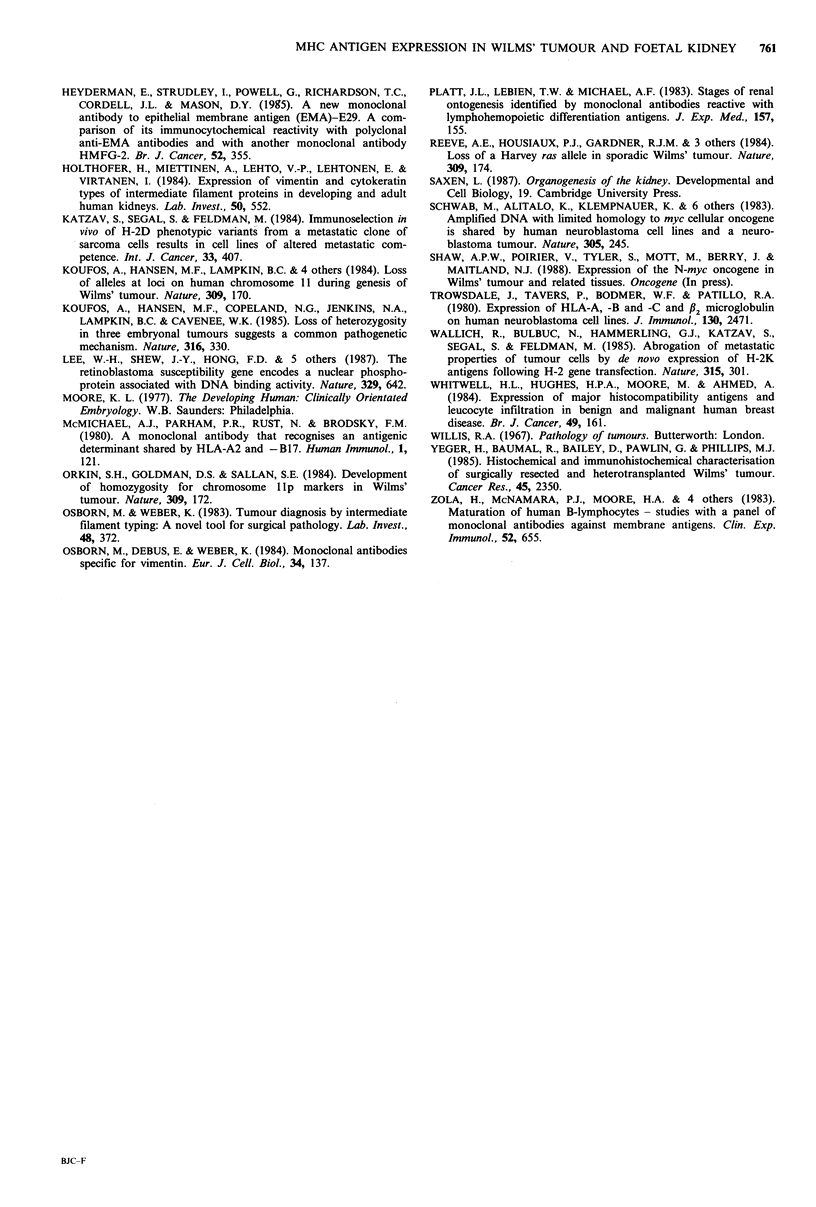# Expression of class I and II major histocompatibility complex antigens in Wilms' tumour and normal developing human kidney.

**DOI:** 10.1038/bjc.1988.303

**Published:** 1988-12

**Authors:** G. M. Borthwick, L. Hughes, C. H. Holmes, S. J. Davis, G. M. Stirrat

**Affiliations:** Department of Obstetrics and Gynaecology, Bristol Maternity Hospital, UK.

## Abstract

**Images:**


*Br. J. Cancer* (1988), **58**, 753–761 © The Macmillan Press Ltd., 1988

### Expression of class I and II major histocompatibility complex antigens in Wilms' tumour and normal developing human kidney

G.M. Borthwick, L. Hughes, C.H. Holmes, S.J. Davis & G.M. Stirrat

*Department of Obstetrics and Gynaecology, Bristol Maternity Hospital, Bristol BS2 8EG, UK.*

**Summary** The Wilms' tumour is a solid childhood tumour of the kidney, consisting of blastema, tubules and mesenchyme. Embryonic tumours, such as Wilms', may arise as a result of a developmental disturbance in differentiation. The expression of class I and II major histocompatibility complex (MHC) antigens was investigated on 6 Wilms' tumours and related to that in the developing human kidney in this immunohistological study, using a panel of monoclonal antibodies.

The Wilms' tumour blastemal cells were class I MHC antigen negative, but differentiated structures were positive. Class II MHC antigens were not observed in Wilms' tumours. In the developing human kidney class I MHC antigen expression was observed on glomeruli from 8 weeks and on tubules from 13 weeks gestational age. Class II MHC antigen expression was observed on glomeruli from 11 weeks and on tubules from 13 weeks gestation. These results suggest that the blastemal cells within the Wilms' tumour may reflect an early stage of development with respect to the expression of MHC antigens.

The expression of major histocompatibility complex (MHC) antigens has been widely studied on many types of tumours, including colonic, breast, gynaecological and renal neoplasms (Csiba *et al.*, 1984; Whitwell *et al.*, 1984; Ferguson *et al.*, 1985; Heineman *et al.*, 1987). These studies have attempted to relate MHC antigen expression to the level of inflammatory cell infiltrate within the tumour. These investigations are frequently based on the proposition that such MHC antigens may generate a host anti-tumour response. However, no clear cut relationship between MHC antigen expression and the inflammatory cell infiltrate has been consistently demonstrated. Thus the significance of MHC antigen expression in this context remains unclear.

In addition to their classical immunological functions, there is now evidence that MHC antigens may also be implicated in normal tissue development and in the process of cellular transformation (Brickell *et al.*, 1983; Platt *et al.*, 1983).

MHC antigen expression on tumour cells arising in adults can be readily related to the MHC antigen status of their normal cellular counterparts. However, in the study of embryonic tumours that arise in children such comparative studies are more complex, since the ontogeny of MHC antigen expression during the normal development of many tissues is unknown.

The Wilms' tumour is a solid childhood kidney tumour containing undifferentiated tissue, that is thought to correspond to early embryonic renal blastema, tubules and mesenchyme (Willis, 1967). Several studies have suggested that mutant alleles are present on chromosome 11 of Wilms' tumours (Koufos *et al.*, 1984; Orkin *et al.*, 1984; Reeve *et al.*, 1984; Fearon *et al.*, 1984) and that the normal gene product of this locus may be involved in normal kidney differentiation (Koufos *et al.*, 1985). These studies support the general theory that embryonic tumours occurring in children may arise as a developmental disturbance in specific cellular differentiation pathways within a given tissue (Willis, 1967).

In this study we have investigated immunohistologically the expression of class I and class II MHC antigens on six Wilms' tumours, childhood healthy kidney and kidney at different stages of development, using a panel of monoclonal antibodies. The expression of MHC antigens on the Wilms' tumours has been related to that expressed in the developing kidney, with particular reference to the stage in development reached by the Wilms' tumour before development is arrested.

Correspondence: G.M. Borthwick.
Received 30 April 1988; and in revised form, 8 July 1988.

### Materials and methods

### *Tissue*

Wilms' tumours (6 cases) and adjacent normal childhood kidney were obtained from therapeutic nephrectomies. The pathology reports confirmed the diagnosis of Wilms' tumour.

Foetal kidney at various stages of development (6–18 weeks) gestation (approximate post-conceptional age) was obtained from ectopic pregnancies and therapeutic terminations of pregnancy. A total of 37 foetal kidneys were studied; 33 kidneys of 6–13 weeks gestation and 4 mid-gestational kidneys (14 at 6–7 weeks, 9 at 8–9 weeks, 5 at 10–11 weeks, 5 at 13 weeks, 2 at 17 weeks and 2 at 18 weeks). The gestational age of the foetal kidney material was determined from foetal footlength (Moore, 1977). Local ethical committee permission was obtained to collect these samples.

Adult kidney was obtained within a few hours of death at post-mortem.

Blocks were taken of all tumour and tissue samples, snap frozen and stored in liquid nitrogen.

### *Immunohistology*

Cryostat sections (5 $\mu$m) were cut, air dried and fixed in acetone for 10 min. An indirect immunoperoxidase method was used to stain the sections. Serial sections were incubated with the monoclonal antibodies listed in Table I for 45 min. The slides were then washed in Tris buffered saline pH 7.6 (TBS). The antibody binding was detected by incubating the sections for 30 min with peroxidase conjugated rabbit anti-mouse immunoglobulin (1/50), (Dako Ltd.) in 10% normal human serum/TBS and then washed in TBS. The sections were then incubated with the peroxidase substrate diaminobenzidine tetrahydrachloride (6 mg in 10 ml TBS and 25 $\mu$l 3% $H_2O_2$ per 10 ml), (Sigma). The reaction was stopped by washing the slides in tap water. The sections were counterstained with haematoxylin, dehydrated, cleared in Histoclear (National Diagnostics) and mounted in DPX mountant. The whole procedure was performed at room temperature.

### Results

### *Class I MHC antigen expression on Wilms' tumour*

All of the Wilms' tumours investigated in this study expressed the classical lobular pattern of islands of epithelial tumour cells surrounded by stroma. Five of the tumours

**Table I** Monoclonal antibodies

| *Monoclonal antibody* | *Specificity* | *Dilution* | *Reference* | *Source* |
|---|---|---|---|---|
| W6/32 | HLA A, B, C, | 1/500 | Barnstable *et al.* (1978) | Sera-Lab. |
| FMC 16 | $\beta_2$ microglobulin | 1/10 | Zola *et al.* (1983) | Sera-Lab. |
| Mel | HLA B27, B7, B22 | 1/200 | Ellis *et al.* (1982) | Gift of A.J. McMichael |
| Ma2.1 | HLA A2, B17 | 1/200 | McMichael *et al.* (1980) | Gift of A.J. McMichael |
| NFK-1 | HLA DP, DQ, DR | 1/500 | Fuggle *et al.* (1983) | Gift of S.V. Fuggle |
| Epithelial membrane antigen (EMA) | Human milk fat globule membrane protein | 1/10 | Heyderman *et al.* (1985) | Dako Ltd. |
| Leu M3 | Monocyte/macrophage | 1/10 | Dimitriu-Bona *et al.* (1983) | Becton-Dickinson |
| F10-89-4 | Leucocyte common antigen | 1/500 | Dalchau *et al.* (1984) | Serotec |
| Factor VIII | Factor VIII related antigen | 1/10 | — | Dako Ltd. |
| Vimentin | Vimentin | 1/10 | Osborn *et al.* (1984) | Amersham |
| Cytokeratin | Cytokeratin | 1/10 | Osborn & Weber (1983) | Amersham |

showed epithelial differentiation but in one specimen there was no epithelial differentiation. In addition, the degree of differentiation varied within the individual tumours.

The stromal component and cells within the stromal matrix of all the tumours expressed class I MHC antigens, as detected by staining with the monoclonal antibodies for the monomorphic and polymorphic regions of class I MHC antigens (W6/32, FMC16, Mel and Ma2.1) (Figure 1a,b). The class I MHC antigen expressing cells within the stromal matrix had a similar distribution to cells expressing the leucocyte common antigen, detected by the monoclonal antibody F10-89-4, and the macrophage antigen, detected by the monoclonal antibody Leu M3. The endothelium vascularising the tumours and identified by the monoclonal antibody to factor VIII, also consistently expressed class I MHC antigens.

The undifferentiated blastema in all the tumours showed no expression of class I MHC antigens (Figure 1a,b). However, of particular interest was the expression of MHC class I antigens on the epithelial elements within the tumour (Figure 1a,b,c). There was no expression of class I MHC antigens on the blastemal component of any of the specimens (Figure 1a,b). However, there was a complex distribution of MHC antigen antibody binding in areas of tubular and glomerular differentiation (Figure 1a,b,c). Some tubules were MHC antigen class I positive while others were clearly negative. Some of the class I MHC antigen positive tubules also expressed epithelial membrane antigen (EMA), a molecule known to be expressed by distal but not proximal tubules in the normal adult kidney (Yeger *et al.*, 1985), while others did not. None of the class I MHC antigen negative tubules expressed EMA.

Further tubular heterogeneity was apparent using monoclonal antibodies to intermediate filament proteins. A monoclonal antibody to cytokeratin bound to all elements within these tumours including blastema and all tubular elements (Figure 2a). Tubular structures showed relatively increased binding of the cytokeratin monoclonal antibody used in this study (Figure 2a). The monoclonal antibody to vimentin also bound to blastemal cells and some but not all tubules (Figure 2b). Some tubular structures also clearly expressed both vimentin and cytokeratin. EMA expressing structures were a subset of the vimentin negative tubular structures. The class I MHC antigen expressing tubular structures did not express vimentin; all vimentin negative tubular structures appeared to express class I MHC antigens. Glomerular structures consistently expressed MHC class I antigens, vimentin and cytokeratin.

*Class II MHC antigen expression on Wilms' tumour*

Class II MHC antigens were not expressed on any of the undifferentiated or differentiated epithelial tumour cells or the stromal matrix. Class II MHC antigen expressing cells were present throughout the tumours (Figure 3); these cells had a similar distribution to those expressing the leucocyte common antigen and the macrophage antigen detected by the monoclonal antibody Leu M3.

*Class I MHC antigen expression on adult kidney and healthy kidney from Wilms' tumour patients*

The expression of class I MHC antigens observed on healthy kidney adjacent to the Wilms' tumour (Figure 4) and normal adult kidney in this study was consistent with previously published reports (Daar *et al.*, 1984a; Fuggle *et al.*, 1983). The healthy kidney from Wilms' tumour cases had a similar class I MHC antigen expression to that seen in the adult kidney. All structures (proximal and distal convoluted tubules, glomeruli, collecting ducts and blood vessels) present in the kidneys investigated in this study consistently expressed class I MHC antigens. The staining intensity for class I MHC antigens in healthy kidney from Wilms' tumour cases varied between structures. The staining was very strong on the glomeruli and interstitial cells in comparison to that observed on tubular structures.

*Class II MHC antigen expression on adult kidney and healthy kidney from Wilms' tumour patients*

The expression of class II MHC antigens on kidney specimens investigated in this study (Figure 5) was consistent with previously published reports (Daar *et al.*, 1984*b*; Fuggle *et al.*, 1983). The class II MHC antigens expression was heterogeneous; glomeruli (endothelium and mesangium) expressed class II MHC antigens in all the adult and healthy kidney from Wilms' tumour patients. The expression of class II MHC antigens on the tubules was very variable between specimens.

*Class I MHC antigen expression in developing human kidney*

Class I MHC antigen expression was investigated in the developing human kidney at varying stages, the earliest specimens of gestational age 6–7 weeks contained collecting ducts and tubules but not glomeruli; at this stage in development these structures did not express class I MHC antigens. A few class I MHC antigen expressing cells were present between the developing structures, these cells had a similar distribution to those detected by the monoclonal antibody to the leucocyte common antigen.

A few developing glomeruli were observed in the kidney at approximately 8 weeks gestational age. Although the degree of differentiation of the glomeruli varied within the specimen. Relatively undifferentiated glomeruli did not express class I MHC antigens whereas differentiated glomeruli expressed class I MHC antigens. At this stage in development of the kidney there were also class I MHC antigen expressing cells present between the developing structures; these cells remain on through development. It is interesting to note that the collecting ducts and tubules did not express class I MHC antigens at this stage in development.

This pattern of class I MHC antigen expression persisted until 12 weeks of gestation. At ~13 weeks gestational age class I MHC antigen expression was also observed on a few (~10%) tubular structures (Figure 6). This expression was very weak in comparison to that observed in the childhood kidney (Figure 4). By ~18 weeks of gestation this weak

**Figure 1** Areas of Wilms' tumour stained for class I MHC antigens with the monoclonal antibody W6/32, which showed (a) differentiated class I MHC antigen expressing structures (arrows) within islands of negative blastemal cells ( × 40), (b) rosetting structures which did not express Class I MHC antigens ( × 40) (arrows) and (c) a class I MHC antigen expressing structure ( × 400).

**Figure 2** Wilms' tumour stained on serial sections with monoclonal antibodies to the intermediate filament proteins (a) cytokeratin (×40) and (b) vimentin (×40). All cells within the tumour expressed cytokeratin; the intensity of staining was increased on differentiated structures. All the cells within the tumour expressed vimentin, apart from a few tubular structures. A few tubular structures expressed both vimentin and cytokeratin.

**Figure 3** Class II MHC antigen expression, detected by the monoclonal antibody NFK-1 on Wilms' tumour (×40). The tumour cells, differentiated and undifferentiated, were class II MHC antigen negative. Class II MHC antigen expressing cells were present throughout the tumour; these cells had a similar distribution to those expressing the leucocyte common antigen and a macrophage marker.

**Figure 4** Class I MHC antigen expression on an area of healthy kidney taken from a Wilms' tumour patient, detected by the monoclonal antibody W6/32 (×40). All cells present in the kidneys investigated expressed class I MHC antigens.

**Figure 5** Class II MHC antigen expression on an area of healthy kidney taken from a Wilms' tumour patient, detected by the monoclonal antibody NFK-1 (×40). Class II MHC antigen expression was present on all glomeruli, the expression on tubular structures was variable between specimens.

**Figure 6** Class I MHC antigen expression on a developing kidney of 13 weeks gestational age, detected using the monoclonal antibody W6/32 (×40). Class I MHC antigen expression was observed on the developing glomeruli, interstitial cells and a few tubular structures (arrows).

**Figure 7** Class I MHC antigen expression on a developing kidney of 18 weeks gestational age, detected using the monoclonal antibody W6/32. (a) Class I MHC antigen expression was observed on interstitial cells and ~25% of tubules (×40) (arrows) and (b) Class I MHC antigen expression on a tubule (×400).

expression of Class I MHC antigens increased to ~25% of tubular structures (Figure 7a,b). A subset of these class I MHC antigen positive tubular structures also expressed the antigen detected by the monoclonal antibody EMA. Between 13 and 18 weeks gestation the majority of developing glomeruli expressed class I MHC antigens.

### *Class II MHC antigen expression in developing human kidney*

Until approximately 11 weeks gestation all structures within the developing kidney did not express class II MHC antigens. At this stage some but not all of the developing glomeruli expressed class II MHC antigens (Figure 8a), the tubular structures were class II MHC antigen negative.

By ~13–18 weeks gestation, however, a few tubular structures (<10% by 18 weeks) clearly expressed class II MHC antigens (Figure 8b). Although this staining pattern was very weak in comparison to that seen in the childhood kidney (Figure 5). As with class I MHC antigens a subset of the class II MHC antigen expressing tubular structures expressed the antigen detected by the monoclonal antibody EMA, which is thought to detect distal tubules and collecting ducts in the adult kidney. Class II MHC antigen expression was still observed on some of the developing glomeruli and on interstitial cells at a gestational age of 13–18 weeks. Class II MHC antigen expressing interstitial cells which were present from a gestational age of 8 weeks had a similar distribution to cells detected by the monoclonal antibody to the leucocyte common antigen and cells expressing the macrophage antigen detected by the monoclonal antibody Leu M3.

### Discussion

The classical picture of the development of the kidney is that the tubules are derived from the metanephric mass of mesoderm (Saxen, 1987). Thus the metanephric tubule becomes an epithelial structure. The glomeruli are also derived from the metanephric blastema. The collecting ducts in the normal kidney are derived from the ureteric bud. The differentiation pathway of cells within the developing kidney can be followed by investigating the expression of the

**Figure 8** Class II MHC antigen expression detected by the monoclonal antibody NFK-1 on developing kidney of (a) 11 weeks (×40) and (b) 18 weeks gestational age (×40). A few class II MHC antigen expressing interstitial cells were present at 11 weeks. Also, at this stage some of the developing glomeruli expressed class II MHC antigens. At 18 weeks gestational age class II MHC antigens were detected on interstitial cells, developing glomeruli and ~10% of tubules.

intermediate filaments vimentin and cytokeratin, which are expressed by mesenchymal and epithelial cells respectively (Bachmann *et al.*, 1983; Holthofer *et al.*, 1983). In this study we have followed the differentiation of structures within the developing kidney by studying the expression of class I and II MHC antigens. Class I MHC antigens are not expressed on glomeruli until 8 weeks, and on tubular structures until 13 weeks gestational age. The class II MHC antigen expression on foetal kidney develops later than class I MHC antigens on glomeruli, at 11 weeks gestational age, but at approximately the same gestational age of 13 weeks on tubular structures. Thus, a clear onset of the expression of these antigens was observed. However, the significance of the timing of this onset and the function of the MHC antigens observed at this stage in development remains unclear.

The majority of cells within the Wilms' tumours comprise undifferentiated blastemal cells. These cells did not express either class I or II MHC antigens. This suggests that the majority of tumour cells may reflect a very early stage of differentiation with respect to the expression of MHC antigens. Class I MHC antigen expression was observed on some but not all of the differentiated tubular and glomerular structures within the tumours. Class II MHC antigen expression was not observed on any of the differentiated structures. This pattern of MHC antigen expression is consistent with the possibility that the tubular like structures, which are a heterogeneous population within the tumour, have differentiated to a varying degree with respect to class I MHC antigen expression. Some of the tubular structures within the Wilms' tumours may reflect an earlier stage of differentiation than others. This is similar to the pattern seen in the developing kidney of structures at different stages of development.

This heterogeneity of antigen expression on differentiating structures in Wilms' tumours can also be detected using monoclonal antibodies to the intermediate filament proteins (Denk *et al.*, 1985; Yeger *et al.*, 1985; Altmannsberger *et al.*, 1984). The class I MHC antigen expressing tubules observed in this study of Wilms' tumours express cytokeratin alone. The tubular structures in this present study which do not express class I MHC antigens co-express cytokeratin and vimentin. This differential expression of intermediate filaments may reflect, along with the class I MHC antigen expression, the progression along the normal differentiation pathway of the tubules within the Wilms' tumour. The most mature tubules express class I MHC antigens and cytokeratin, a subset of these express EMA which is a marker for distal convoluted tubules and collecting ducts. The tubules in the kidney are derived from the metanephric mass of mesoderm, which accounts for their expression of vimentin (Saxen, 1987). The metanephric tubule develops into an epithelial structure, and during this phase, co-expression of cytokeratin and vimentin may be observed. When cytokeratin alone is expressed by the tubule, it has become an epithelial structure, and it is at this stage that class I MHC antigen expression is observed.

The corresponding expression of class II MHC antigens between Wilms' tumours and developing kidney was not observed. The differentiated structures within the Wilms' tumours were class II MHC antigen negative. Thus, whereas glomeruli and tubules in normal foetal kidney expressed class II MHC antigens from approximately 11 and 13 weeks respectively, the structures within the Wilms' tumours were invariably negative. Thus, class II MHC antigen expression on Wilms' tumours did not reflect that observed in normal developing kidney. The significance of this lack of class II MHC antigen expression is not understood.

There is now evidence that as well as having an immune function, MHC antigens may have a non-immune function (Edidin, 1983). MHC antigens are thought to play a role in cellular recognition and interaction. It is difficult to perceive, although it is possible that MHC antigens detected on the structures of developing foetal kidney have a classical immunological role. However, it may be that in this situation the MHC antigens are necessary for cell communication and thus for normal development to occur.

The metastatic properties of some tumours may be related to the level of expression of class I MHC antigens (Katzav *et al.*, 1984). Transfection of the mouse MHC class I gene into a mouse fibrosarcoma abolished the metastatic properties of the tumour (Wallich *et al.*, 1985); the tumour associated antigens presented along with MHC class I antigens may facilitate a host response. Studies on neuroblastoma have shown that the tumour has low levels of expression of class I MHC antigens and high levels of expression of the *myc* gene family (Trowsdale *et al.*, 1980; Schwab *et al.*, 1983). It has been proposed that there is a relationship between the level of N-*myc* and class I MHC antigen expression (Bernards *et al.*, 1986); transfer of the N-*myc* gene into a rat neuroblastoma cell line led to a decrease in the class I MHC antigen expression, increased the growth rate and increased the metastatic capability of the cells. Studies on Wilms' tumours by Northern blotting and *in situ* hybridisation of mRNA have shown that there are elevated levels of N-*myc* in this tumour type, located in the blastemal cells (Shaw *et al.*, 1988). The study presented here shows that these blastemal cells do not express MHC antigens. It may be that in Wilms' tumours N-*myc* inhibits the expression of class I MHC antigens on the blastemal cells, thus maintaining their metastatic capabilities.

A recent report has demonstrated that the retinoblastoma susceptibility gene encodes a nuclear phosphoprotein associated with DNA binding activity (Lee *et al.*, 1987). This gene is absent in retinoblastoma; normally the gene product regulates other genes within the cell, which implies that it is necessary for normal development to occur. This evidence supports the theory that childhood tumours arise from a disturbance along the normal developmental pathway (Willis, 1967).

In this study we have clearly demonstrated that the majority of cells within the Wilms' tumour may reflect an early stage of differentiation with respect to the expression of MHC antigens. These results, therefore, support the theory that embryonic tumours arise as a result of a disturbance in specific cellular differentiation.

This study was supported by grants from the Cancer and Leukaemia in Childhood Trust, the Wellcome Trust and the Cancer Research Campaign. We would like to thank Dr J. Berry, Bristol Maternity Hospital and Dr M. Mott, Bristol Childrens Hospital for their help with this study.

### References

ALTMANNSBERGER, M., OSBORN, M., SCHAFER, H., SCHAUER, A. & WEBER, K. (1984). Distinction of nephroblastomas from other childhood tumours using antibodies to intermediate filaments. *Virchows Arch. B. Cell. Pathol.*, **45**, 113.

BACHMANN, S., KRIZ, W., KUHN, C. & FRANKE, W.W. (1983). Differentiation of cell types in the mammalian kidney by immunofluorescence microscopy using antibodies to intermediate filament proteins and desmoplakins. *Histochemistry*, **77**, 365.

BERNARDS, R., DESSAIN, S.K. & WEINBERG, R.A. (1978). N-*myc* amplification causes down-modulation of MHC class I antigen expression in neuroblastoma. *Cell*, **47**, 667.

BARNSTABLE, C.J., BODMER, W.F., BROWN, G. & 4 others (1978). Production of monoclonal antibodies to group A erythrocytes, HLA and other human cell surface antigens – new tools for genetic analysis. *Cell*, **14**, 9.

BRICKELL, P.M., LATCHMAN, D.S., MURPHY, D., WILLISON, K. & RIGBY, P.W.J. (1983). Activation of a Qa/Tla class I major histocompatibility antigen gene is a general feature of oncogenesis in the mouse. *Nature*, **306**, 756.

CSIBA, A., WHITWELL, H.L. & MOORE, M. (1984). Distribution of histocompatibility and leucocyte differentiation antigens in normal human colon and in benign and malignant colonic neoplasms. *Br. J. Cancer*, **50**, 699.

DAAR, A.S., FUGGLE, S.V., FABRE, J.W., TING, A. & MORRIS, P.J. (1984*a*). The detailed distribution of HLA-A,B,C antigens in normal human organs. *Transplantation*, **38**, 287.

DAAR, A.S., FUGGLE, S.V., FABRE, J.W., TING, A. & MORRIS, P.J. (1984*b*). The detailed distribution of MHC class II antigens in normal human organs. *Transplantation*, **38**, 293.

DALCHAU, R., KIRKLEY, J. & FABRE, J.W. (1980). Monoclonal antibody to a human leucocyte-specific membrane glycoprotein probably homologous to the leucocyte-common (L-C) antigen of the rat. *Eur. J. Immunol.*, **10**, 737.

DENK, H., WEYBORA, W., RATSCHEK, M., SOHAR, R. & FRANKE, W.W. (1985). Distribution of vimentin, cytokeratins and desmosomal-plaque proteins in human nephroblastoma as revealed by specific antibodies: Co-existence of cell groups of different degrees of epithelial differentiation. *Differentiation*, **29**, 88.

DIMITRIU-BONA, A., BURMESTER, G.R., WATERS, S.J. & WINCHESTER, R.J. (1983). Human mononuclear phagocyte differentiation antigens. I. Patterns of antigenic expression on the surface of human monocytes and macrophages defined by monoclonal antibodies. *J. Immunol.*, **130**, 145.

EDIDIN, M. (1983). MHC antigens and non-immune functions. *Immunol. Today*, **4**, 269.

ELLIS, S.A., TAYLOR, C. & McMICHAEL, A.J. (1982). Recognition of HLA-B27 and related antigens by a monoclonal antibody. *Human Immunol.*, **5**, 49.

FEARON, E.R., VOGELSTEIN, B. & FEINBERG, A.P. (1984). Somatic deletion and duplication of genes on chromosome 11 in Wilms' tumours. *Nature*, **309**, 176.

FERGUSON, A., MOORE, M. & FOX, H. (1985). Expression of MHC products and leucocyte differentiation antigens in gynaecological neoplasms: An immunohistological analysis of the tumour cells and infiltrating leucocytes. *Br. J. Cancer*, **52**, 551.

FUGGLE, S.V., ERRASTI, P., DAAR, A.S., FABRE, J.W., TING, A. & MORRIS, P.J. (1983). Localisation of major histocompatibility complex (HLA-ABC and DR) antigens in 46 kidneys. *Transplantation*, **35**, 385.

HEINEMANN, D., SMITH, P.J.B. & SYMES, M.O. (1987). Expression of histocompatibility antigens and characterisation of mononuclear cell infiltrates in human renal cell carcinomas. *Br. J. Cancer*, **56**, 433.

HEYDERMAN, E., STRUDLEY, I., POWELL, G., RICHARDSON, T.C., CORDELL, J.L. & MASON, D.Y. (1985). A new monoclonal antibody to epithelial membrane antigen (EMA)–E29. A comparison of its immunocytochemical reactivity with polyclonal anti-EMA antibodies and with another monoclonal antibody HMFG-2. *Br. J. Cancer*, **52**, 355.

HOLTHOFER, H., MIETTINEN, A., LEHTO, V.-P., LEHTONEN, E. & VIRTANEN, I. (1984). Expression of vimentin and cytokeratin types of intermediate filament proteins in developing and adult human kidneys. *Lab. Invest.*, **50**, 552.

KATZAV, S., SEGAL, S. & FELDMAN, M. (1984). Immunoselection *in vivo* of H-2D phenotypic variants from a metastatic clone of sarcoma cells results in cell lines of altered metastatic competence. *Int. J. Cancer*, **33**, 407.

KOUFOS, A., HANSEN, M.F., LAMPKIN, B.C. & 4 others (1984). Loss of alleles at loci on human chromosome 11 during genesis of Wilms' tumour. *Nature*, **309**, 170.

KOUFOS, A., HANSEN, M.F., COPELAND, N.G., JENKINS, N.A., LAMPKIN, B.C. & CAVENEE, W.K. (1985). Loss of heterozygosity in three embryonal tumours suggests a common pathogenetic mechanism. *Nature*, **316**, 330.

LEE, W.-H., SHEW, J.-Y., HONG, F.D. & 5 others (1987). The retinoblastoma susceptibility gene encodes a nuclear phosphoprotein associated with DNA binding activity. *Nature*, **329**, 642.

MOORE, K. L. (1977). *The Developing Human: Clinically Orientated Embryology*. W.B. Saunders: Philadelphia.

McMICHAEL, A.J., PARHAM, P.R., RUST, N. & BRODSKY, F.M. (1980). A monoclonal antibody that recognises an antigenic determinant shared by HLA-A2 and –B17. *Human Immunol.*, **1**, 121.

ORKIN, S.H., GOLDMAN, D.S. & SALLAN, S.E. (1984). Development of homozygosity for chromosome 11p markers in Wilms' tumour. *Nature*, **309**, 172.

OSBORN, M. & WEBER, K. (1983). Tumour diagnosis by intermediate filament typing: A novel tool for surgical pathology. *Lab. Invest.*, **48**, 372.

OSBORN, M., DEBUS, E. & WEBER, K. (1984). Monoclonal antibodies specific for vimentin. *Eur. J. Cell. Biol.*, **34**, 137.

PLATT, J.L., LEBIEN, T.W. & MICHAEL, A.F. (1983). Stages of renal ontogenesis identified by monoclonal antibodies reactive with lymphohemopoietic differentiation antigens. *J. Exp. Med.*, **157**, 155.

REEVE, A.E., HOUSIAUX, P.J., GARDNER, R.J.M. & 3 others (1984). Loss of a Harvey *ras* allele in sporadic Wilms' tumour. *Nature*, **309**, 174.

SAXEN, L. (1987). *Organogenesis of the kidney*. Developmental and Cell Biology, 19. Cambridge University Press.

SCHWAB, M., ALITALO, K., KLEMPNAUER, K. & 6 others (1983). Amplified DNA with limited homology to *myc* cellular oncogene is shared by human neuroblastoma cell lines and a neuroblastoma tumour. *Nature*, **305**, 245.

SHAW, A.P.W., POIRIER, V., TYLER, S., MOTT, M., BERRY, J. & MAITLAND, N.J. (1988). Expression of the N-*myc* oncogene in Wilms' tumour and related tissues. *Oncogene* (In press).

TROWSDALE, J., TAVERS, P., BODMER, W.F. & PATILLO, R.A. (1980). Expression of HLA-A, -B and -C and $\beta_2$ microglobulin on human neuroblastoma cell lines. *J. Immunol.*, **130**, 2471.

WALLICH, R., BULBUC, N., HAMMERLING, G.J., KATZAV, S., SEGAL, S. & FELDMAN, M. (1985). Abrogation of metastatic properties of tumour cells by *de novo* expression of H-2K antigens following H-2 gene transfection. *Nature*, **315**, 301.

WHITWELL, H.L., HUGHES, H.P.A., MOORE, M. & AHMED, A. (1984). Expression of major histocompatibility antigens and leucocyte infiltration in benign and malignant human breast disease. *Br. J. Cancer*, **49**, 161.

WILLIS, R.A. (1967). *Pathology of tumours*. Butterworth: London.

YEGER, H., BAUMAL, R., BAILEY, D., PAWLIN, G. & PHILLIPS, M.J. (1985). Histochemical and immunohistochemical characterisation of surgically resected and heterotransplanted Wilms' tumour. *Cancer Res.*, **45**, 2350.

ZOLA, H., McNAMARA, P.J., MOORE, H.A. & 4 others (1983). Maturation of human B-lymphocytes – studies with a panel of monoclonal antibodies against membrane antigens. *Clin. Exp. Immunol.*, **52**, 655.